# Annotated Spanish general election debate transcriptions 1993-2023

**DOI:** 10.1038/s41597-025-05818-8

**Published:** 2025-09-29

**Authors:** Fermín L. Cruz, Fernando Enríquez, F. Javier Ortega, José A. Troyano

**Affiliations:** Department of Computer Languages and Systems, University of Seville, Seville, Spain

**Keywords:** Politics, Communication

## Abstract

The automatic analysis of political discourse requires systematic resources, particularly for non-Anglophone electoral debates, which differ from parliamentary sessions. This paper describes the DebatES dataset, containing manually reviewed transcriptions of nearly all Spanish nationally televised general election debates since 1993. Transcripts are segmented into turns and thematic blocks and include participant metadata. The data is enriched with linguistic-stylistic metrics derived from NLP analysis and extensive annotations combining Large Language Models and manual validation. Annotations cover turn topics, emotions, relevant entity mentions, electoral proposals, and factual claims. To facilitate access and reuse, the dataset is distributed in standard formats (XML and CSV), accompanied by interactive reports for visual exploration. This dataset provides a valuable resource for researchers in linguistics, political science, and language technologies, opening new avenues for studying the evolution of discourse, persuasive strategies, and ideological polarisation within the Spanish political context.

## Background & Summary

In recent years, the automatic analysis of political discourse has gained particular relevance due to the growing interest in understanding political phenomena from an interdisciplinary perspective, combining linguistics, political science, and language technologies^1,2^. Electoral and parliamentary debates are key spaces for studying how political ideas are articulated, how candidates and parties establish their positions on issues of public relevance, and how they interact in scenarios of discursive confrontation. Having extensive and systematic linguistic resources that compile detailed transcriptions of these debates allows for more precise investigation of aspects such as the evolution of political discourse, ideological polarization, argumentative strategies, the emotional impact of political language, or the communicative behavior of specific political actors. In this context, various datasets have emerged in multiple languages, formats, and analytical objectives, especially focusing on the 20th and 21st centuries, although their geographical and linguistic distribution remains uneven. Among the most important resources with transcriptions of electoral debates are mainly datasets in English concerning US elections, such as *USA Presidential Debates*^3^ and *Tidy US Debate Transcripts*^4^, both with broad historical coverage (1960–2020) and enriched metadata, as well as the French dataset *FREDSum*, which offers televised presidential debates with argumentative summaries. Within the domain of political debate, but moving to transcriptions of parliamentary sessions instead of televised debates, the multilingual parallel corpus *Europarl*^5^, with speeches from the European Parliament, and the *ParlaMint*^6^ project, with recent debates (2015–2022) from 29 national European parliaments, linguistically enriched for comparative analysis, stand out. Other relevant resources include the British historical corpus *Hansard Corpus*^7^, the Swedish parliamentary corpus *RixVox*^8^, with audio-text alignment (2003–2023), and the Norwegian parliamentary corpus^9^, with transcriptions and participant metadata (1945–2024). Although these datasets provide great value to the study of political discourse, English predominates, highlighting the need to expand similar resources to other languages and regions. Furthermore, most of these resources focus on parliamentary sessions, with those specifically centered on electoral debates still being scarce. The type of communication in electoral debates presents substantially different characteristics: while parliamentary debates are usually oriented towards formal legislative discussion with more structured and predictable institutional dynamics, electoral debates tend to be direct, strategic, and emotionally intense discursive confrontations, aimed at capturing the immediate attention of the voter and defining clear ideological positions against political adversaries.

The DebatES dataset aims to be a tool to facilitate analysis of political information, especially the messages that political parties send to their potential voters in the weeks leading up to an election. The resource contains processed, enriched, and annotated textual transcriptions of almost all electoral debates broadcast in Spain over the last 30 years (only two debates have not been included, as recordings were not publicly available). Televised electoral debates in Spain began in 1993 with the confrontation between the leaders of the two main parties, the Spanish Socialist Workers’ Party (PSOE) and the People’s Party (PP). After a long absence, they returned in 2008, also featuring debates between the candidates of these two parties. Since then, they have been a key element in election campaigns, with formats varying in the number of participants and rules. Starting in 2015, the debates expanded to include more than two political parties, given the emergence of new political parties with significant electoral impact.

Our resource includes transcriptions of the debates segmented into turns, which are grouped into thematic blocks. Additionally, linguistic-stylistic metrics are included at the turn and participant level, as well as annotations of topics discussed, emotions, mentions of entities, proposals and claims found in each turn. The data is provided in two formats: XML files containing all information at the debate level, and CSV files with the different types of information structured as relational tables. In addition to these formats designed for computational processing, we include interactive reports in HTML format, with graphs, sections, hyperlinks, filters, etc., allowing users without technical knowledge to comfortably navigate the entire resource and perform searches and analyses of the debate content.

The transcriptions of the electoral debates that form the basis of this resource have also been used in the context of an annotation task focused on logical fallacies^10^. Given the inherently interpretative and subjective nature of this type of annotation, these fallacy labels are not included as part of the distributed dataset. However, they illustrate the potential of the resource for supporting more nuanced and theory-driven analyses of argumentative discourse in electoral settings.

## Methods

The resource includes transcriptions of the electoral debates, as well as three types of annotations: 1) descriptive information about the debates and their participants, 2) linguistic information resulting from applying natural language processing (NLP) tools, annotations from the political domain obtained automatically and manually reviewed, and 3) manual annotations.

### Transcription and Text Structuring

Based on the publicly available videos for each electoral debate, transcription was carried out using *WhisperX*^11^, an advanced speech recognition model that includes speaker diarization. All the debates between candidates for Spain’s General Elections, held on nationally broadcast radio or television up to the 2023 General Elections, were included, with the exception of two debates for which access to recordings was unavailable: one from 1993 and another from 2015. Note that debates were not held during every election cycle.

Subsequently, the transcriptions were manually reviewed and corrected to ensure content fidelity. Furthermore, information about the participants, including their full names and corresponding political affiliation, was incorporated manually. Each debate was segmented into turns, assigning each one to its respective speaker.

It should be noted that the transcription process inevitably results in the loss of some prosodic and interactional information, such as pauses and interruptions. In the particular case of overlapping speech, which frequently occur in the heat of a debate, the transcriptions render these concurrent utterances sequentially. Throughout this process, every effort was made to remain as faithful as possible to the original speech turns.

### Linguistic Processing

For text processing, we used the *spaCy* library^12^, employing the transformer-based model for Spanish (es_dep_news_trf). This process allowed for sentence segmentation, text tokenization, and the extraction of morphosyntactic information, from which the following linguistic metrics were calculated at the turn and participant level for each debate:

- Type-Token Ratio (ttr): Measures the vocabulary richness in the text. It is calculated by dividing the number of unique lemmas by the total number of tokens in the text. Higher values indicate a more varied use of words.
- Stopword Proportion (stop_ratio): Represents the percentage of words that are stopwords, indicating the level of informational content in the text.
- Average Sentence Length (avg_sent_len): Indicates the average number of words per sentence, reflecting the structural complexity of the discourse.
- Average Dependencies per Verb (avg_dep_per_verb): Measures the average number of words that depend on a verb in the syntactic structure, which can reflect syntactic complexity.
- Punctuation Proportion in Text (punct_ratio): Indicates the relative amount of punctuation marks, which can reflect text segmentation.
- Adjective Proportion (adj_ratio): Shows what percentage of the text is composed of adjectives, reflecting the descriptive level of the content.
- Adverb Proportion (adv_ratio): Measures the presence of adverbs in the text, which can indicate a more detailed or nuanced style.

In addition to including these metrics in the data files for each debate, at the participant and turn level, the resource independently includes all the raw information obtained from the linguistic processing, including syntactic dependency trees. This information can be useful for conducting other types of linguistic studies on the debate texts.

### Automatic Annotation using LLMs and Manual Review

To enrich the resource with semantic and discursive information, the texts were processed using the language models *Gemini 2.0 Flash* (https://cloud.google.com/vertex-ai/generative-ai/docs/models/gemini/2-0-flash) and *Gemini 2.5 Pro* (https://cloud.google.com/vertex-ai/generative-ai/docs/models/gemini/2-5-pro). The researchers used preview versions of the models called gemini-2.0-flash-exp and gemini-2.5-pro-exp-03-2, available through Google AI Studio. After numerous proof-of-concept tests, different prompts were selected to automatically obtain the various annotations. The prompts and models used for each type of information are included in the code repository^13^. The types of annotations generated through this mechanism are as follows:

- Thematic blocks per debate: Segmentation of the debate into thematic units, with summary titles. These blocks are introduced by the debate moderator; only these moderator turns were processed using the language model to find the blocks.
- Topics per turn: Generation of a title that synthesizes the topics discussed throughout the turns. Each topic can span one or more consecutive turns, so the list of topics serves as a thematic index for each debate, facilitating access to turns on a specific topic.
- Relevant mentions per turn: Identification of references in the text of turns to political entities, institutions, economic indicators, social issues, and other relevant elements in the political context. Definitions for each type can be found in Table 1.
- Proposals per turn: Extraction of electoral promises or references to the electoral program made by candidates in each turn.
- Claims per turn: Identification of statements based on data, statistics, or references to past or present facts, which could be subject to a fact-checking process.
- Emotions per sentence: Identification of emotions found in each sentence according to Ekman’s scale of six basic emotions^14^ (joy, sadness, fear, surprise, anger, and disgust). This includes both emotions expressed by the speaker and those evoked in the receiver (e.g., if a message might generate fear or surprise in the recipient).

All automatically generated information was manually reviewed, discarding any errors found. Additionally, manual annotation of a sample of turns was carried out to measure the coverage of the automatic annotations performed. Section validation describes this entire process and shows the metrics obtained.

**Table 1:** Types of entities mentioned in DebatES.

Type	Definition
Person	Names of public figures, such as national and international politicians, journalists, economists, etc.
Organization	Public and private organizations, associations, and institutions. Includes governing bodies, administrations, and companies, among others.
Location	Geographical and administrative places including cities, countries, regions, and other points of interest around the world, including buildings or specific addresses within cities. Also includes mentions related to locations, such as languages or demonyms, among others.
Economy	Mentions of aspects related to the economy, such as growth, employment, investment, financial policy, social protection. Also includes fiscal issues: taxes, reforms, and fiscal justice.
Ideology	Terms describing different schools of thought, movements, and political positions, such as communism, liberalism, and socialism. Also covers political movements and parties, like the pro-independence left and Catalan republicans, as well as concepts related to the organization of the state and society, such as self-determination and constitutionalism. Also includes terms related to each of these currents or political positions, in the sense that they are elements championed by these movements or that generate discord between antagonistic positions.
Law	Legal norms and laws regulating various aspects of social, political, and economic life in a country. They include constitutions, statutes of autonomy, specific laws on topics like education, health, equality, and human rights, as well as penal and civil codes, and international treaties. In addition to direct mentions, references to proposals, judicial processes, application, and consequences of laws are included.
Media	Media outlets, media groups, television and radio programs, digital platforms, and entities related to the dissemination of information.
Party	Names of political parties, coalitions, and political formations that have existed or currently exist, primarily in the Spanish context.
Public & Social Issues	Includes terms related to social welfare and the development of citizenship through essential public services and support policies. Covers health, education, transportation, social protection, security, and infrastructure, as well as policies on equality, labor rights, access to housing, pension system, immigration, work-life balance, poverty reduction, and quality of life. Mentions of problems related to these issues are included.

## Data Records

The dataset is available from the Figshare^15^. The resource is distributed in two formats adapted for computational use:

- XML: one XML file for each debate, containing all transcriptions and annotations made on them.
- CSV: one CSV file for each type of information, processed and organized to facilitate their querying and analysis.

Additionally, a series of interactive HTML reports are included, allowing manual consultation of the information. Finally, the complete outputs of the linguistic processing of the texts are included.

The following sections describe each of these formats.

### XML Format

**Fig. 1:**
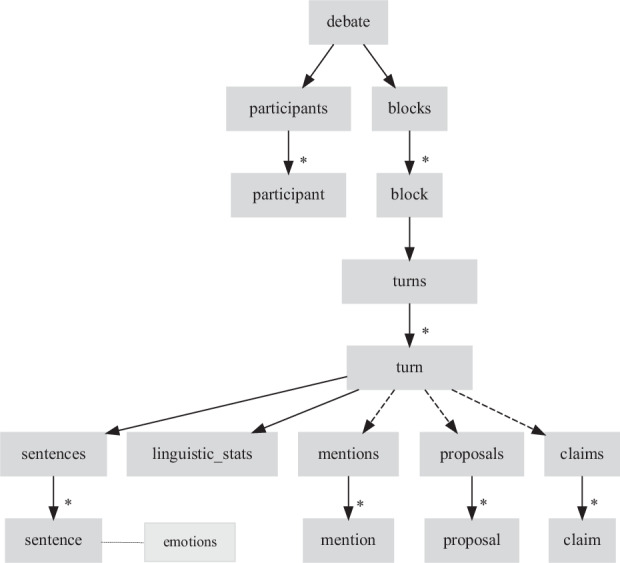
XML scheme for each debate. Dashed lines indicate optionality (the parent may or may not have that child). The asterisk (*) indicates that there can be one or more occurrences of the corresponding element. The dotted line indicates that *emotions* is an attribute of the *sentence* element.

All information related to each debate is included in an XML file, whose name contains the date the debate was held (see Table 2). The hierarchical structure of these XML files is shown in Fig. 1. Below, the elements of this structure and the attributes included in each are described; note that all elements contain a id attribute with a unique identifier, which has been omitted in the description:

- **1. Debate**: The root element debate includes attributes with metadata about the debate:
- date: Date the debate was held.
- election-date: Date of the election corresponding to this debate.
- media: Media outlet that organized the debate.
- **2. Participants**: Each participant has the following attributes:
- full-name: Full name of the participant.
- party: Political party represented by the participant.
- Detailed linguistic statistics: Includes attributes for lexical richness (ttr), ratio of function words (stop-ratio), average sentence length (avg-sent-len), average dependencies per verb ratio (avg-dep-per-verb), punctuation ratio (punct-ratio), adjective ratio (adj-ratio), adverb ratio (adv-ratio), and average syntactic dependency distance (avg-dep-dist).
- **3. Blocks**: Each block represents a central theme of the debate, introduced by the moderator. The topic attribute contains a short textual description of the main theme of the block.
- **4. Turns**: Each turn corresponds to a speaking turn and contains the following attributes:
- participant-id: Participant identifier, matching the id of one of the participants listed at the beginning of the XML.
- topic: Main topic of the turn.
- **5. Sentences**: Each sentence corresponds to the text of one sentence within a participant’s turn, such that the entire text of the turn can be reconstructed from the complete sequence of sentence elements. The emotions attribute indicates the emotions detected in that sentence, according to Ekman’s scale of six basic emotions^14^ (joy, sadness, fear, surprise, anger, and disgust).
- **6. Mentions**: Each mention corresponds to an entity or indicator referenced by the participant during their turn, and contains the following attributes:
- type: Type of entity or indicator mentioned, from the following options: LOCATION, ORGANIZATION, PARTY, MEDIA, POLITICIAN, JOURNALIST, LAW, ECONOMIC_INDICATOR, IDEOLOGY, SOCIAL_ISSUE, and PUBLIC_SERVICE. Definitions for each type can be found in Table 1.
- text: Segment of text from the turn that constitutes the mention.
- **7. Proposals**: Each proposal corresponds to an electoral promise made by the participant in their turn. The text contained in each proposal does not correspond to a literal segment extracted from the turn, but rather is a summary of the proposal.
- **8. Claims**: Each claim corresponds to an assertion made by the participant in their turn about data or facts related to the past or near present, which could be subject to a fact-checking process. The text contained in each claim does not correspond to a literal segment extracted from the turn, but rather is a summary of said assertion.

**Table 2:** Debates for Spanish General Elections in DebatES.

Elect.	Date	Participants	Organizer
1993	24 May	J.M. Aznar (PP), F. González (PSOE)	Antena 3
2008	25 Feb.	M. Rajoy (PP), J.L.R. Zapatero (PSOE)	AcademiaTV
	3 March	M. Rajoy (PP), J.L.R. Zapatero (PSOE)	AcademiaTV
2011	7 Nov.	M. Rajoy (PP), A.P. Rubalcaba (PSOE)	AcademiaTV
	23 Nov.	P. Iglesias (Podemos), A. Rivera (Cs)	Univ.Carlos III
2015	30 Nov.	P. Sánchez (PSOE), P. Iglesias (Podemos), A. Rivera (Cs)	El País
	14 Dec.	P. Sánchez (PSOE), M. Rajoy (PP)	Atresmedia-AcademiaTV
2016	13 June	M. Rajoy (PP), P. Sánchez (PSOE), P. Iglesias (UP), A. Rivera (Cs)	AcademiaTV
	16 April	C. Álvarez de Toledo (PP), M.J. Montero (PSOE), I. Montero (UP), I. Arrimadas (Cs), G. Rufián (ERC-Sobiranistes), A. Esteban (PNV)	RTVE
2019 (April)	20 April	T.García Egea (PP), F. Sicilia (PSOE), A. Garzón (UP), T. Cantó (Cs), G. Rufián (ERC-Sobiranistes), L. Borras (JxCAT), A. Esteban (PNV)	LaSexta
	22 April	P. Sánchez (PSOE), P. Casado (PP), P. Iglesias (UP), A. Rivera (Cs)	RTVE
	23 April	P. Sánchez (PSOE), P. Casado (PP), P. Iglesias (UP), A. Rivera (Cs)	Atresmedia
	1 Nov.	A. Lastra (PSOE), C. Álvarez de Toledo (PP), I. Arrimadas (Cs), I. Montero (UP), I. Espinosa de los Monteros (Vox), G. Rufián (ERC), A. Esteban (PNV)	RTVE
2019 (Nov.)	2 Nov.	F. Sicilia (PSOE), C. Gamarra (PP), M. Rodríguez (Cs), N. Vera (UP), J.O. Smith (Vox), G. Rufián (ERC), A. Esteban (PNV), L. Borras (JxCAT)	LaSexta
	4 Nov.	P. Sánchez (PSOE), P. Casado (PP), P. Iglesias (UP), A. Rivera (Cs), S. Abascal (Vox)	AcademiaTV
	7 Nov.	M.J. Montero (PSOE), A. Pastor (PP), I. Arrimadas (Cs), I. Montero (UP), R. Monasterio (Vox)	LaSexta
	10 July	P. Sánchez (PSOE), A.N. Feijóo (PP)	Atresmedia
2023	13 July	P. López (PSOE), C. Gamarra (PP), A. Vidal (Sumar), I. Espinosa de los Monteros (Vox), A. Esteban (PNV), O. Matute (EH Bildu), G. Rufián (ERC)	RTVE
	19 July	P. Sánchez (PSOE), Y. Díaz (Sumar), S. Abascal (Vox)	RTVE

### CSV Format

The dataset is divided into 9 CSV files, each representing a specific type of information (debates, participants, turns, etc.). To link the information between the different files, identifier columns are used:

- debate_id: Unique identifier assigned to each debate during processing (generally based on the original filename). Allows linking all data belonging to the same debate across the different files.
*_xml_id: Identifiers such as participant_xml_id, block_xml_id, turn_xml_id, sentence_xml_id, etc., that correspond to the original identifiers present in the debate XMLs. Allow maintaining the original hierarchical relationships within the context of a single debate.

The purpose and columns of each CSV file are detailed below.

- **1. debates.csv**: Stores the general information for each processed debate. Each row represents one debate, including these columns:
- debate_id: Unique identifier for the debate. **Primary key** for this file and **foreign key** in others.
- date: Date the debate took place (YYYY-MM-DD format).
- election_date: Date of the elections related to the debate (YYYY-MM-DD format).
- media: Name of the media outlet that organized or broadcast the debate.
- **2. participants.csv**: Contains information about each participant in each debate, including their *aggregated* linguistic statistics for that entire debate. The columns included are:
- debate_id: Links to debates.csv.
- participant_xml_id: Original participant ID. Useful for linking with turns.csv within the same debate_id.
- full_name: Full name of the participant.
- party: Name of the political party the participant belongs to.
- ttr: Type-Token Ratio (lexical diversity) of the participant in the entire debate.
- stop_ratio: Proportion of function words (stop words) used by the participant.
- avg_sent_len: Average length of sentences spoken by the participant.
- avg_dep_per_verb: Average number of syntactic dependencies per verb.
- punct_ratio: Proportion of punctuation marks in the participant’s speech.
- adj_ratio: Proportion of adjectives used by the participant.
- adv_ratio: Proportion of adverbs used by the participant.
- avg_dep_dist: Average distance between dependent words in the syntactic tree.
- **3. blocks.csv**: Lists the thematic blocks into which each debate was divided, including these columns:
- debate_id: Links to debates.csv.
- block_xml_id: Original block ID. Useful for linking with turns.csv within the same debate_id.
- topic: The topic or title of the block.
- **4. Turns.csv**: Contains each turn of speech by a participant, including linguistic statistics *specific* to that turn. Acts as a central table to connect participants, blocks, sentences, mentions, etc. The columns are:
- debate_id: Links to debates.csv.
- block_xml_id: Links to blocks.csv, indicating which thematic block the turn belongs to.
- turn_xml_id: Original turn ID. **Primary key** (along with debate_id) for linking with sentences, mentions, proposals and claims.
- participant_xml_id: Links to participants.csv, indicating who made the turn.
- topic: The specific topic discussed in this turn.
- ttr, stop_ratio, avg_sent_len, avg_dep_per_verb, punct_ratio, adj_ratio, adv_ratio, avg_dep_dist: Linguistic statistics calculated *only for the text of this turn*.
- **5. sentences.csv**: Stores each individual sentence spoken during the turns, including these columns:
- debate_id: Links to debates.csv.
- turn_xml_id: Links to turn.csv, indicating which turn this sentence belongs to.
- sentence_xml_id: Original sentence ID.
- text: The textual content of the sentence.
- emotions: Detected emotions associated with the sentence (if any). May contain multiple emotions separated by commas (e.g., happiness,anger). If none, the field will be empty.
- **6. mentions.csv**: Records all mentions of named entities (people, parties, places, etc.) made during each turn, including these columns:
- debate_id: Links to debates.csv.
- turn_xml_id: Links to turns.csv, indicating in which turn the mention was made.
- mention_xml_id: Original mention ID.
- type: The type of entity mentioned (e.g., PERSON, PARTY, LOCATION, MEDIA, ORGANIZATION, IDEOLOGY, PUBLIC_SOCIAL_ISSUES, etc.).
- text: The exact text of the mention made.
- **7. proposals.csv**:Lists the specific policy or action proposals made during an turn, including these columns:
- debate_id: Links to debates.csv.
- turn_xml_id: Links to turns.csv.
- proposal_xml_id: Original proposal ID.
- text: The descriptive text of the proposal.
- **8. claims.csv**: Contains the factual assertions or claims made by participants during their turns, including these columns:
- debate_id: Links to debates.csv.
- turn_xml_id: Links to turns.csv.
- claim_xml_id: Original claim ID.
- text: The text of the claim made.

### Interactive HTML Reports

Interactive reports in HTML format are included in the resource, allowing visualization of all annotated information at the debate, participant, and political party level, and providing convenient access to turns related to each annotation. The reports also allow filtering of the displayed information by debates, participants and keywords. These reports are accessed through the index.html file located in the report folder. This file can be opened with any modern web browser. The reports can also be consulted online at https://defalac.github.io/DebatES-report/.

**Fig. 2:**
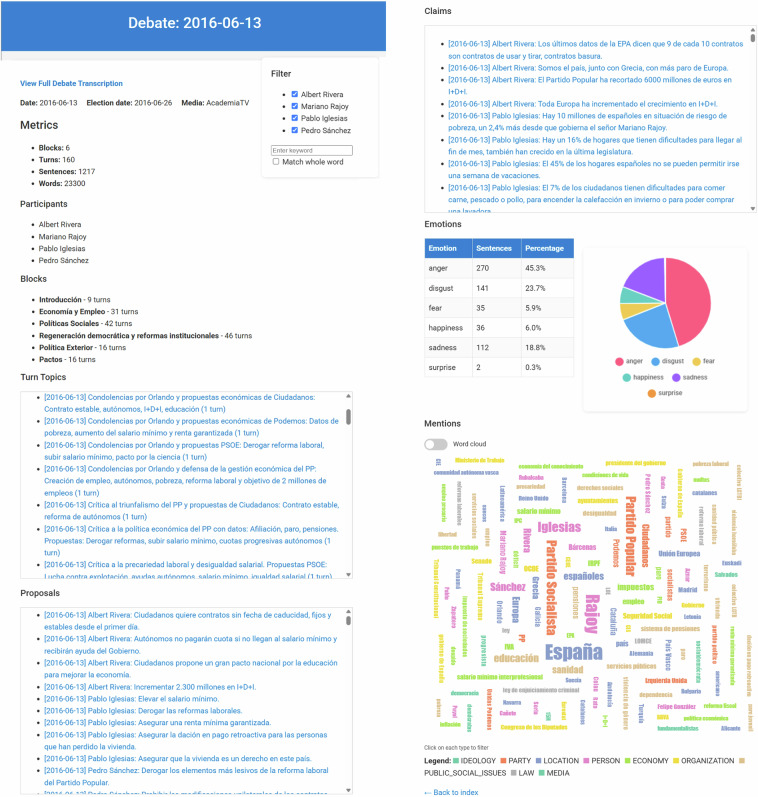
Screenshots of a debate-level report.

Figure 2 shows some screenshots of a debate-level report. The report begins by displaying metadata and key metrics about the debate, along with a link to access the full transcript. It then presents the thematic blocks into which the debate is divided. Following this, the report showcases the topics of turns, proposals and claims. Each of these elements is linked to the specific point in the transcript where they appear.

A floating filter panel allows users to filter turns by participant or by specific keywords. When filters are applied, the report updates to display only the topics, proposals and claims relevant to the selected turns.

Finally, the report includes graphical representations of emotions (shown as both a table and a pie chart) and of different types of mentions. These visualizations reflect only the currently displayed turns based on the applied filters. Mentions can be visualized either as a word cloud (as shown in figure 2) or as a bar chart, depending on the selected view. Additionally, users can filter which types of mentions are displayed.

Similar reports at both party and participant levels are included, aggregating all turns by each party or participant in the dataset.

### Linguistic Processing Information Dump

For each debate, a CSV file is included with the output from the *spaCy* linguistic analyzer when processing all the texts from the transcriptions. For each word or token in the text, morphological and syntactic information is included. The columns of each CSV are as follows:

- debate_date: Date of the debate.
- turn_id: The identifier of the turn within the debate.
- sentence_id: The identifier of the sentence within the turn.
- token_id: The position of the token within the text (its index).
- token_text: The original text of the token as it appears in the document.
- lemma: The base or canonical form of the token (e.g., “run” instead of “running”).
- pos: Part of speech — the grammatical category of the token (noun, verb, adjective, etc.).
- dep_head_index: The index of the token’s syntactic head (the word it depends on).
- dep_head_text: The text of the token’s syntactic head.
- dep_relation: The type of syntactic relation between the token and its head (e.g., subject, object, modifier).
- is_stop: Indicates whether the token is a stop word (common word like “the”, “and”, “of”).
- is_alpha: Indicates whether the token consists only of alphabetic characters (no numbers or punctuation).

## Technical Validation

The creation of the DebatES resource required combining automatic, semi-automatic, and manual processes, thus a careful technical validation process was carried out to ensure the quality, precision, and coverage of the generated information.

The initial automatic transcription was performed using the *WhisperX* model^11^, which has demonstrated high accuracy in automatic speech recognition and speaker diarization in various contexts. However, considering the relevance of the discursive content and its potential use for academic research, all automatically generated transcriptions were manually reviewed and corrected. This review allowed resolving common inaccuracies such as errors in specific words, proper nouns, technical terms, or interruptions and overlaps in speaking turns, thereby ensuring the fidelity and total accuracy of the final text included in the resource.

Regarding linguistic processing using the *spaCy* tool^12^, we used the transformer-based model for Spanish (es_dep_news_trf), whose performance reported by the developers achieves an accuracy greater than 95% in extracting syntactic dependency relations and greater than 99% in the other analysis tasks on which our metrics are based. These accuracy levels ensure the robustness and reliability of the calculated linguistic metrics, allowing for reliable subsequent analyses on syntactic complexity, lexical richness, or communicative style.

With respect to semantic annotations obtained via Large Language Models (LLMs), although these techniques offer high general performance, they present specific risks such as hallucinations or content errors. Therefore, a rigorous subsequent manual review process was established to validate the automatically generated annotations:

- *Thematic blocks*: The thematic segmentation generated by the LLM was fully reviewed manually, making occasional adjustments in cases where the boundaries between thematic blocks were unclear or when the title synthesis did not sufficiently reflect the content of the turns.
- *Topics*: Recent studies, such as those presented in the Hallucination Evaluation Leaderboard (https://huggingface.co/spaces/vectara/Hallucination-evaluation-leaderboard), report an accuracy for the used model gemini-2.5-pro close to 100% in tasks of generating headlines for news using similar LLMs, a context comparable to this specific task. As an additional measure, we reviewed the correctness of the topics assigned to a stratified sample of turns, totaling 378 sentences, sufficient to guarantee a 95% confidence interval and a 5% margin of error. The accuracy obtained in this evaluation was 93%.
- *Mentions, proposals, and claims*: A complete manual validation of the automatically generated annotations was carried out, eliminating incorrect or incomplete ones. Furthermore, based on the same previous sample of turns, we performed manual annotation of all mentions, proposals, and claims to estimate the coverage of the automatic annotations, obtaining coverage values of 90%, 100%, and 83%, respectively. However, there is a possibility that certain relevant mentions, proposals, or claims may not have been identified by the model, so we recommend using this data as a facilitating tool, not an exhaustive one, for discourse analysis.
- *Emotions*: Manual annotation was performed on the same sample of 378 sentences to estimate the precision and recall of the automatically annotated emotions, yielding an F1-micro score of 0.95. As this is a multi-label classification task, True Positives, False Positives, and False Negatives were accumulated for each label, and from these, precision and recall were calculated, which were then used to estimate the F1-micro score. This result allows us to state that, although the annotations are not free from occasional inaccuracies, they exhibit sufficient precision to be useful when used in aggregate, although we recommend caution in their use for highly sensitive analyses or those dependent on absolute accuracy.

## Data Availability

All code used for linguistic processing, automatic annotations using language models (including prompts), and the generation of interactive reports in HTML format is available in GitHub^13^.
